# Treatment of heart failure related cardiogenic shock: Time to fill the evidence gap

**DOI:** 10.1002/ehf2.15335

**Published:** 2025-06-02

**Authors:** Janine Pöss, Anne Freund, Holger Thiele

**Affiliations:** ^1^ Heart Center Leipzig at Leipzig University and Leipzig Heart Science Leipzig Germany

Cardiogenic shock is the leading cause of death in hospitalized patients with acute myocardial infarction.^1^ Whereas the majority of cardiogenic shock causes were related to acute myocardial infarction in the past, heart failure is now emerging to be the most frequent underlying aetiology in several contemporary registries.^2^, ^3^, ^4^, ^5^, ^6^ The SHARC criteria are important for the underlying aetiology of cardiogenic shock.^7^

Until recently, the only evidence‐based treatment strategy proven to reduce the still unacceptably high mortality of infarct‐related cardiogenic shock is revascularization of the infarct related coronary artery.^8^, ^9^, ^10^ Disappointingly, other treatment strategies such as inotropes, vasopressors, intraaortic balloon pumping (IABP),^11^ or venoarterial extracorporeal membrane oxygenation (VA‐ECMO) have failed to prove a mortality benefit in randomized clinical trials in the infarct‐related shock setting.^12^, ^13^ Recently, a percutaneous microaxial flow pump showed a mortality reduction in selected ST‐elevation myocardial infarction (STEMI) patients without risk of hypoxic brain injury.^14^ In an individual patient data, meta‐analysis of all nine randomized trial comparing mechanical circulatory support (MCS) versus control pointed out that routine MCS does not reduce mortality while increasing complications. Only the subgroup of STEMI patients without risk of hypoxic brain injury benefited from MCS.^15^

The low number of available trials is partly related to the fact that randomized trials in the acute shock setting are difficult to perform and multiple trials have been stopped because of slow enrolment. Furthermore, due to the heterogeneity of the cardiogenic shock population, the risk of neutral results is high.^2^, ^16^, ^17^ So far, only 10 randomized trials with an inclusion rate >100 patients have been completed with the vast majority focusing on infarct‐related cardiogenic shock.^2^, ^13^, ^14^ The only relevant randomized trial in heart failure‐related cardiogenic shock is the recently published Altshock‐2 trial, which failed to show a benefit of IABP in this setting.^18^ This trial was based on observational data, which have suggested that IABP support could lead to greater haemodynamic effects than in patients with infarct‐related cardiogenic shock with clinical stabilization and improved tissue perfusion.^19^ As a consequence, there was the believe that IABP may serve as a bridge to durable left ventricular assist device implantation or heart transplantation. However, the neutral Altshock‐2 results once again underline the necessity of randomized trial evidence (*Figure* 1).

**Figure 1:**
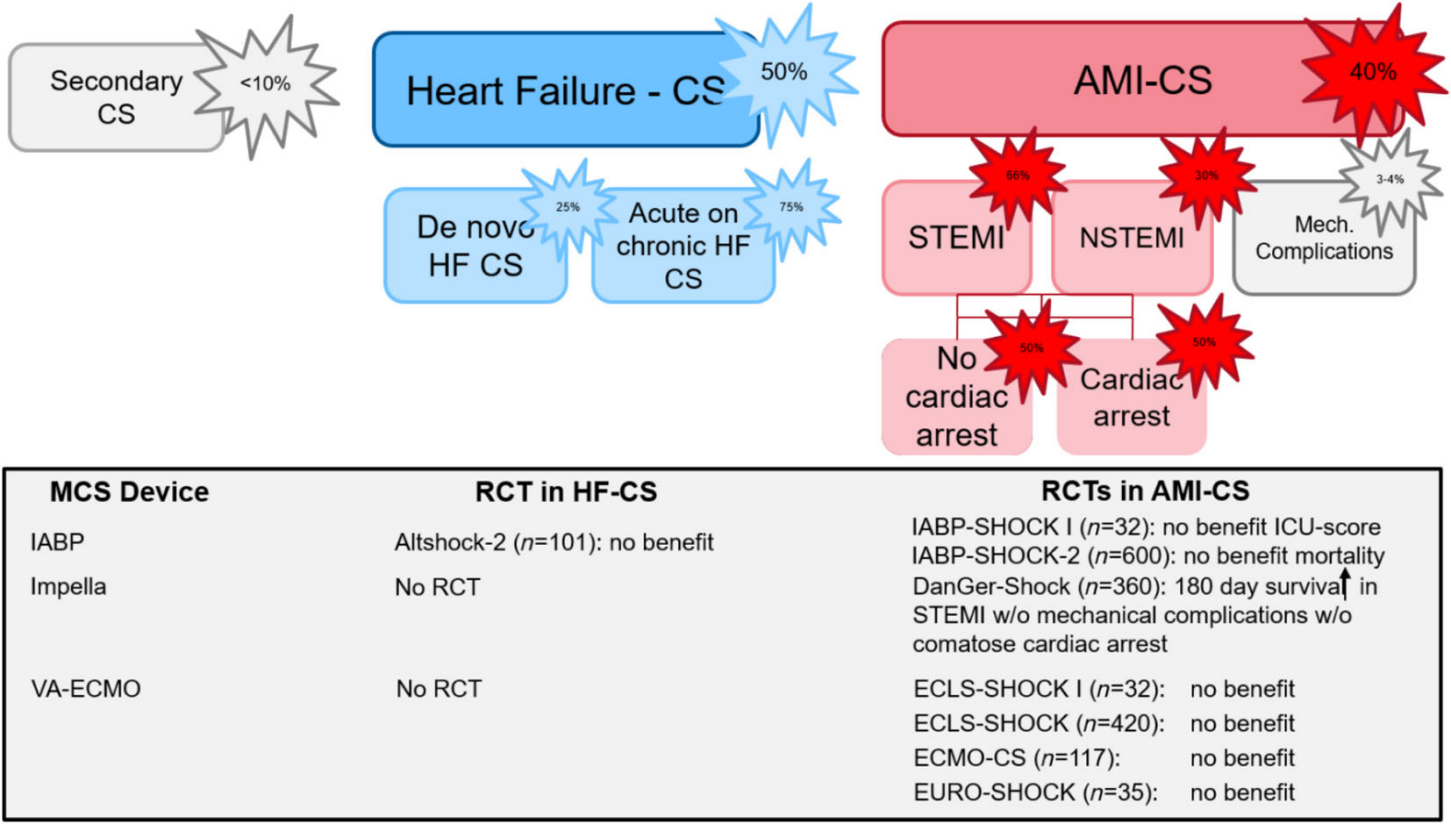
Incidence of different cardiogenic shock aetiologies based on SHARC criteria and randomized evidence for mechanical circulatory support. AMI, acute myocardial infarction; CS, cardiogenic shock; HF, heart failure; IABP, intraaortic balloon pump; MCS, mechanical circulatory support; Mech., mechanical; NSTEMI, non‐ST‐elevation myocardial infarction; RCT, randomized controlled trial; STEMI, ST‐elevation myocardial infarction; VA‐ECMO, venoarterial extracorporeal membrane oxygenation.

In the current issue of ESC Heart Failure, Cherbi et al. report data from the FRENSHOCK registry. A total of 772 patients with cardiogenic shock at 49 centres were included between April and October 2016, of which roughly 63.7% had heart failure‐related or non‐ischaemic cardiogenic shock. The predominance of heart failure‐related cardiogenic shock compared with shock related to acute myocardial infarction described in this registry is in line with other recent observational data reported above.^2^, ^3^, ^4^, ^5^, ^6^, ^20^ As the vast majority of cardiogenic shock trials only included patients with infarct‐related cardiogenic shock, the available evidence for the large group of non‐ischaemic shock patients is scarce. This is of relevance because the clinical trajectory of patients with non‐ischaemic and ischaemic shock varies significantly. While presentation of patients with ischaemic shock mostly is highly acute, patients with non‐ischaemic shock are often characterized by a more non‐linear course, switching back and forth between the different shock stages.^21^ Additionally, important differences were described between the two cohorts of patients regarding baseline characteristics (age, comorbidities, incidence of cardiac arrest, echocardiographic and haemodynamic parameters) and regarding the hospital course (use of temporary/durable MCS, bleeding complications and length of hospital stay).^22^

In most of the registries comparing patients with infarct‐related and heart‐failure related cardiogenic shock, 1‐year mortality rates of the latter group was often lower but also substantial reaching more than 40%. Cherni et al. describe short‐term 1‐month and mid‐term 1‐year mortality rates of patients presenting with this aetiology of 25.6% and 45.7%, which is in line with the other reports. Notably, the combined endpoint of 1‐year mortality, heart transplantation or durable ventricular assist device was reached by more than half of the patients (53.9%). Of interest is a multivariate analysis showing five independent factors for 1‐year mortality, which included age, chronic kidney disease, norepinephrine use, active cancer and acute renal replacement therapy. Interestingly, the majority of patients had a maximal SCAI stage C or D, and only 1% was in SCAI stage E. Furthermore, also roughly 9% pre‐shock patients presenting in SCAI stage B were included. The distribution of SCAI stages differs significantly from an analysis of the Critical Care Cardiology Trials Network (CCCTN) registry published by Bhatt et al. in 2021, in which the proportion of patients presenting in SCAI stage E was considerably higher (46% of de novo heart failure cardiogenic shock patients and more than 1/3 of the acute on chronic heart failure shock patients).^23^

Unfortunately, Cherni et al. give no information about whether patients had de novo heart failure‐shock or acute on chronic heart failure cardiogenic shock. This is of importance since again, these two entities show different clinical characteristics. Patients with de novo heart failure (e.g., fulminant myocarditis) mostly present in a hyperacute setting. In contrast, patients with acute on chronic heart failure shock often present less acutely with a rather gradual deterioration of a pre‐existing condition.^6^ According to observational data, the group of acute on chronic heart failure shock patients is much larger than the group of de novo heart failure shock patients.^6^, ^23^ Again, clinical trajectories differ significantly between the two cohorts, with higher rates of cardiopulmonary resuscitation in de novo heart failure patients^24^ and higher rates of heart replacement therapy in the group of acute on chronic heart failure patients.^6^ These diverging characteristics might also explain the observed differences in mortality rates between the two groups: In‐hospital mortality rates are higher in de novo heart failure patients whereas 1‐year mortality rates have been described to be higher in acute on chronic heart failure patients.^6^, ^23^, ^25^

The epidemiological development with increasing rates of non‐ischaemic shock, the substantial clinical differences compared with patients with infarction‐related shock and the non‐negligible mortality rates underline the need to perform dedicated randomized clinical trials including patients with non‐ischaemic shock. Important challenges to be addressed by the trialists include, amongst others, to perform an optimal phenotyping and patient selection out of this highly heterogeneous cohort of patients, to decide whether or not to include patients after cardiopulmonary resuscitation and to select the most appropriate endpoint. If we overcome these hurdles, we will manage to shed at least a little light on the still blind spot.

## Conflict of interest

None declared.
